# 肺癌术后患者下肢深静脉血栓的发生率及相关危险因素分析

**DOI:** 10.3779/j.issn.1009-3419.2023.102.16

**Published:** 2023-05-20

**Authors:** DU Hui, ZHAO Honglin, ZHAO Qingchun, CHEN Jun

**Affiliations:** ^1^075000 张家口，河北北方学院附属第一医院胸外科; ^1^Department of Thoracic Surgery, The First Affiliated Hospital of Hebei North University, Zhangjiakou 075000, China; ^2^300052 天津，天津医科大学总医院肺部肿瘤外科; ^2^Department of Lung Cancer Surgery, Tianjin Medical University General Hospital, Tianjin 300052, China

**Keywords:** 肺肿瘤, 术后, 下肢深静脉血栓, 发生率, 危险因素, Lung neoplasms, Postoperative, Deep venous thrombosis, Incidence, Risk factors

## Abstract

**背景与目的** 肺癌在恶性肿瘤中的发病率和死亡率居首位，已成为当前研究的热点问题。临床上根据病理类型将肺癌分为小细胞肺癌（small cell lung cancer, SCLC）和非小细胞肺癌（non-small cell lung cancer, NSCLC），其中NSCLC又包括腺癌、鳞癌及其他类型肺癌，占所有肺癌的80%左右；静脉血栓栓塞症（venous thromboembolism, VTE）包括下肢深静脉血栓（deep venous thrombosis, DVT）和肺动脉栓塞（pulmonary embolism, PE），是一种在肺癌患者中有极高的发病率和死亡率的并发症。本研究旨在明确肺癌术后患者下肢DVT的发生率，并揭示下肢DVT发生的危险因素。**方法** 选择天津医科大学总医院肺部肿瘤外科2021年12月-2022年12月收治的83例肺癌手术患者，入院及术后均行下肢静脉彩超检查，以分析肺癌患者术后下肢DVT的发生率；对患者的临床病例资料进行统计分析，探讨肺癌术后DVT发生的危险因素；并同期监测肺癌患者术后第1、3、5天的凝血功能和血小板，以研究其对血栓发生患者的观测价值。**结果** 肺癌术后患者中，发生下肢DVT为25例，发生率为30.1%，肺癌手术患者中III期+IV期及年龄>60岁者术后下肢DVT发生率更高，差异具有统计学意义（P=0.031, P=0.028）；肺癌术后血栓患者术后第1、3、5天的D-二聚体均明显高于肺癌术后非血栓患者（P<0.05），而两组纤维蛋白原（fibrinogen, FIB）和血小板间差异无统计学意义（P>0.05）。**结论** 我们中心肺癌术后患者DVT总体发生率为30.1%；术后中晚期、年龄较大患者更易发生DVT；其中，D-二聚体值较高的患者应考虑到VTE事件发生的可能。

## Abstract (en)

**Background and objective** Lung cancer is the first leading cause of morbidity and mortality among the malignant tumors, which has become a hot issue in current research. Clinically, lung cancer is divided into small cell lung cancer (SCLC) and non-small cell lung cancer (NSCLC) according to the pathological types. NSCLC includes adenocarcinoma, squamous cell carcinoma and other types of lung cancer, accounting for about 80% of all lung cancer. Venous thromboembolism (VTE) includes deep venous thrombosis (DVT) and pulmonary embolism (PE), which is a recognized complication in lung cancer patients with higher morbidity and mortality. The aim of this study is to determine the incidence of DVT and reveal the risk factors for DVT in the postoperative patients with lung cancer. **Methods** We collected 83 postoperative patients with lung cancer admitted to the Department of Lung Cancer Surgery, Tianjin Medical University General Hospital from December 2021 to December 2022. All these patients were examined by color Doppler ultrasound of lower extremity vein upon admission and after operation to analyze the incidence of DVT. In order to explore the possible risk factors for DVT in these patients, we further analyzed the correlations between DVT and their clinical features. At the same time, the changes of coagulation function and platelet were monitored to investigate the value of blood coagulation in the patients with DVT. **Results** DVT occurred in 25 patients after lung cancer operation, and the incidence rate of DVT was 30.1%. Further analysis found that the incidences of postoperative lower limb DVT were higher in lung cancer patients of stage III+IV or over 60 years of age (P=0.031, P=0.028). D-Dimer level in patients with thrombosis was significantly higher than that in non-thrombus patients on the 1^st^, the 3^rd^, and the 5^th^ day after operation (P<0.05), and there was no significant difference in platelets and fibrinogen (FIB) (P>0.05). **Conclusion** The overall incidence of DVT in our center after lung cancer patients operation was 30.1%. Late-stage and older postpatients were more likely to develop DVT, and these patients with higher D-Dimer values should be considered the possibility of VTE events.

目前，肺癌在国内恶性肿瘤中的发病率和死亡率居首位，已成为当前研究的热点问题^[1]^。静脉血栓栓塞症（venous thromboembolism, VTE）包括深静脉血栓（deep venous thrombosis, DVT）（主要发生在下肢）和肺动脉栓塞（pulmonary embolism, PE），是一种在肺癌患者中有极高的发病率和死亡率的并发症^[2]^。引起肺癌患者发生VTE的原因除肿瘤自身因素外，还包括肺癌的治疗手段，如手术、中心静脉导管（central venous catheter, CVC）、化疗药物、靶向药物、抗血管生成药物等^[3,4]^。据相关研究^[5,6]^统计，与非手术患者相比，接受手术治疗的肺癌患者DVT的发生风险至少增加2倍，PE的发生风险增加3倍。肺癌术后患者的VTE事件会对其治疗产生严重后果，如辅助治疗延迟、出血风险、血栓的反复发生、生活质量的下降以及总生存期的缩短等。有报道^[7⇓-9]^称，伴有VTE发生的癌症更具有侵袭性。DVT是肺癌术后患者最常见的并发症之一，DVT的栓子一旦脱落形成PE，则对患者预后产生严重不良影响。然而，DVT的发生具有隐匿性，且临床表现多无特异性。肺癌术后患者是DVT的高危人群，本研究对符合纳入标准的肺癌术后患者进行双下肢静脉彩超检查，计算DVT发生率并分析临床资料，旨在明确肺癌术后患者DVT的发生率，并揭示其发生的危险因素。

## 1 资料与方法

### 1.1 临床资料

收集天津医科大学总医院肺部肿瘤外科2021年12月-2022年12月收治的肺癌手术患者83例，患者入院及术后进行双下肢静脉彩超检查，对其DVT的发生率进行统计分析；对肺癌术后患者的临床病例资料进行统计分析，如性别、年龄、身体质量指数（body mass index, BMI）、高血压史、吸烟史、肿瘤类型、肿瘤部位、手术方式、手术持续时间、术中出血量、术后卧床时间、术后住院时间等，探讨肺癌术后患者DVT发生的危险因素；并同期监测肺癌患者术后第1、3、5天的凝血功能和血小板，以研究其对血栓发生患者的观测价值。

### 1.2 纳入与排除标准

纳入标准：（1）最终有明确病理诊断为肺癌，且分期均采用国际抗癌联盟（Union for International Cancer Control, UICC）第8版肺癌肿瘤原发灶-淋巴结-转移（tumor-node-metastasis, TNM）分期标准；（2）入院时进行双下肢静脉彩超检查及凝血功能和血常规检查；（3）术后再次进行双下肢静脉彩超，并于术后第1、3、5天进行凝血功能和血常规检查。排除标准：（1）患者既往有下肢静脉血栓病史或既往下肢手术病史；（2）入院前口服抗凝药或者抗血小板药的患者；（3）入院时存在DVT的患者；（4）临床住院资料不完整的患者。

### 1.3 统计学方法

数据采用SPSS 20.0软件进行统计。计量资料采用t检验，如数据资料不符合正态分布或方差齐性则采用秩和检验；计数资料采用卡方检验。P<0.05为差异具有统计学意义。

## 2 结果

### 2.1 肺癌术后患者DVT的发生率及相关危险因素分析

共纳入符合纳排标准的肺癌手术患者83例，其中术后发生DVT的25例患者归为血栓组，术后未发生DVT的58例患者归为非血栓组，肺癌术后患者DVT的发生率为30.1%。对两组患者临床资料进行卡方检验可知，III期+IV期或年龄>60岁的肺癌患者术后DVT发生率更高，差异具有统计学意义（P=0.031, P=0.028）（表1）。

**表1:** 83例肺癌术后患者血栓组与非血栓组的临床资料分析

Item		DVT group (n=25)	Non-DVT group (n=58)	P
Gender	Male	12 (48.0%)	39 (67.2%)	0.098
	Female	13 (52.0%)	19 (32.8%)	
Age (yr)	≤60	6 (24.0%)	29 (50.0%)	0.028
	>60	19 (76.0%)	29 (50.0%)	
BMI (kg/m^2^)	≤25	14 (56.0%)	36 (62.1%)	0.604
	>25	11 (44.0%)	22 (37.9%)	
Smoking history	No	13 (52.0%)	19 (32.7%)	0.098
	Yes	12 (48.0%)	39 (67.3%)	
Hypertension history	No	12 (48.0%)	33 (56.9%)	0.455
	Yes	13 (52.0%)	25 (43.1%)	
Operation methods	VATS	15 (60.0%)	35 (60.3%)	0.977
	Open surgery	10 (40.0%)	23 (39.7%)	
Operation times	≤4 h	19 (76.0%)	45 (77.6%)	0.875
	>4 h	6 (24.0%)	13 (22.4%)	
Tumor site	Left lung	10 (40.0%)	25 (43.1%)	0.793
	Right lung	15 (60.0%)	33 (56.9%)	
Tumor stage	I+II	10 (40.0%)	38 (65.5%)	0.031
	III+IV	15 (60.0%)	20 (34.5%)	
Pathological types	Adenocarcinoma	19 (76.0%)	35 (60.3%)	0.170
	Others	6 (24.0%)	23 (39.7%)	
Postoperative hospital times	≤7 d	12 (48.0%)	28 (48.2%)	0.982
	>7 d	13 (52.0%)	30 (51.8%)	
Time in the custody room	≤48 h	14 (56.0%)	29 (50.0%)	0.616
	>48 h	11 (44.0%)	29 (50.0%)	
Intraoperative bleeding	≤50 mL	18 (72.0%)	35 (60.3%)	0.311
	>50 mL	7 (28.0%)	23 (39.7%)	
Postoperative blood transfusion	No	15 (60.0%)	42 (72.4%)	0.263
	Yes	10 (40.0%)	16 (27.6%)	
Use hemostatic drugs	No	21 (84.0%)	44 (75.9%)	0.409
	Yes	4 (16.0%)	14 (24.1%)	

DVT: deep venous thrombosis; BMI: body mass index; VATS: video-assisted thoracic surgery.

### 2.2 肺癌术后患者DVT发生与血液指标的关系

肺癌血栓患者术后第1、3、5天的D-二聚体水平均明显高于非血栓患者，差异具有统计学意义（P<0.05）；肺癌血栓患者术后第1、3、5天的纤维蛋白原（fibrinogen, FIB）和血小板与肺癌术后非血栓患者相比，差异无统计学意义（P>0.05）（表2）。

**表2:** DVT组与非DVT组患者凝血指标的分析

Index	Clinical data	DVT group (n=25)	Non-DVT group (n=58)	P
D-Dimer (median)	The 1^st^ day	3,846	985	0.001
	The 3^rd^ day	7,438	1,206	<0.001
	The 5^th^ day	8,748	1,644	0.005
FIB (median)	The 1^st^ day	4.09	3.87	0.620
	The 3^rd^ day	5.55	5.89	0.240
	The 5^th^ day	4.84	5.90	0.232
Platelet (Mean±SD)		248±75	246±80	0.940
FIB: fibrinogen.				

表3列出了25例血栓患者的D-二聚体值，患者血栓发病面积越广泛，则D-二聚体值越高；PE患者的D-二聚体平均值高于DVT患者，DVT患者的D-二聚体值远高于肌间静脉血栓（calf muscle venous thrombosis, CMVT）患者。

**表3:** 25例肺癌患者VTE的发生情况及其D-二聚体水平

Order number	Occurrence of VTE	The 5^th^ day D-Dimer value
1	Bilateral CMVT, bilateral popliteal vein thrombosis and PE	>10,000
2	Bilateral popliteal vein thrombosis and PE	>10,000
3	Right femoral vein thrombosis and PE	>10,000
4	Right tibiofibular vein thrombosis and PE	9,212
5	Bilateral popliteal vein thrombosis with bilateral PE	8,903
6	Bilateral superficial femoral vein thrombosis, bilateral PE	9,903
7	Right popliteal vein thrombosis	6,892
8	Left popliteal vein thrombosis	6,120
9	Left CMVT and left tibiofibular vein thrombosis	5,439
10	Right tibiofibular vein thrombosis	7,239
11	Left CMVT	5,430
12	Left CMVT	4,372
13	Left CMVT	3,580
14	Left CMVT	2,891
15	Left CMVT	4,840
16	Right CMVT	5,740
17	Right CMVT	6,741
18	Right CMVT	6,890
19	Right CMVT	783
20	Right CMVT	675
21	Right CMVT	1,240
22	Right CMVT	3,671
23	Bilateral CMVT	6,650
24	Bilateral CMVT	6,435
25	Bilateral CMVT	2,350

PE: pulmonary embolism; CMVT: calf muscle venous thrombosis.

## 3 讨论

肺癌是目前国内发病率和死亡率最高的恶性肿瘤，是当前研究的热点问题。由于目前微创手术、达芬奇机器人手术及加速康复外科的普遍推广，肺癌术后并发症有所减少，但是DVT（包括CMVT）仍是肺癌患者最常见的并发症之一^[10,11]^。肺癌术后患者的VTE事件会对其治疗产生严重后果，如辅助治疗延迟、出血风险、血栓的反复发生、生活质量的下降以及总生存期的缩短等^[12,13]^。有报道^[7⇓-9]^称，伴有VTE发生的癌症更具有侵袭性，且预后更差；然而，在国内虽然有不少针对肿瘤患者伴发VTE的研究，但是单独对肺癌患者DVT发生因素进行探讨的研究却较少。本研究就肺癌患者术后DVT的发生率及其相关危险因素进行了探究。

本中心肺癌患者术后DVT的发病率为30.1%；肺癌细胞可产生组织因子（tissue factor, TF）、癌性促凝物质（cancer procoagulant, CP）、细胞因子和炎症因子等，从而直接激活凝血^[14,15]^。同时肺癌患者术后下肢DVT的发生率远高于入院时，手术治疗增加了肺癌患者下肢DVT的发生风险，据文献^[16]^报道，与非手术癌症患者相比，外科手术将增加2倍-4倍的术后血栓事件发生风险。术后血栓事件仍然是癌症术后患者死亡的主要原因之一，肺癌术后1个月内血栓的发生率最高，且其风险会持续到术后3个月^[17]^。手术引起肺癌患者发生血栓的原因包括：外科操作造成大量的血管损伤而直接激活凝血纤溶系统；术中长时间的特定体位和术后长时间的卧床造成静脉血液瘀滞；肺组织减少引起的缺氧状态，肿瘤的不完全切除，术后使用止血药物、抗血管生成药物、靶向药物和术前D-二聚体水平升高等因素都会增加术后血栓的发生风险^[18]^。

本研究结果提示，分期为III期+IV期或年龄>60岁的肺癌患者术后下肢DVT发生率更高；相关研究^[19]^显示，肺癌的组织学类型及分期与血栓的发生密切相关，其中NSCLC患者血栓的发生率高于SCLC患者；肺腺癌患者血栓的发生率高于肺鳞状细胞癌患者。晚期肿瘤则是VTE发生的独立高危因素。同时患者的自身因素对血栓的形成也有重要影响，如：高龄状态、既往存在VTE病史、外周静脉置管、制动状态、创伤、口服避孕药、妊娠、C反应蛋白升高、D-二聚体水平升高、可溶性P-选择素升高、BMI≥35 kg/m^2^、抗磷脂抗体升高、化疗前血小板计数异常或白细胞计数异常等^[20]^。

本研究结果提示，肺癌患者术后发生下肢DVT的患者术后第1、3、5天的D-二聚体明显高于肺癌术后无DVT的患者（P<0.05），而两组间FIB和血小板间无统计学差异（P>0.05）。由此可见，肺癌手术患者凝血纤维溶解系统处于紊乱状态。D-二聚体也可以很好地监测术后伴有DVT发生的血栓患者，从而进一步指导抗凝治疗。2021年美国胸外科医师协会（American College of Chest Physicians, ACCP）发布的第11版肿瘤患者VTE防治指南^[21]^指出，有效的预防和治疗可以降低恶性肿瘤患者VTE的发生率并提高其生存率，并建议住院癌症患者和接受大型手术患者应采取预防措施。低分子肝素（low molecular weight heparin, LMWH）被认为是预防和治疗癌症患者发生VTE的首选方案^[22,23]^，但应注意出血风险。
